# ﻿Taxonomic status and nomenclature of *Cephalotaxuslanceolata* (Cephalotaxaceae)

**DOI:** 10.3897/phytokeys.222.101974

**Published:** 2023-03-24

**Authors:** Yong Yang

**Affiliations:** 1 Co-Innovation Center for Sustainable Forestry in Southern China, Key Laboratory of State Forestry and Grassland Administration on Subtropical Forest Biodiversity Conservation, College of Biology and Environment, Nanjing Forestry University, 159 Longpan Road, Nanjing 210037, China Nanjing Forestry University Nanjing China

**Keywords:** Cephalotaxaceae, *Cephalotaxuslanceolata*, gymnosperms, nomenclature, the Shenzen Code

## Abstract

The nomenclature of *Cephalotaxuslanceolata* is controversial. After a thorough literature investigation, the nomenclatural problems have been resolved. This name was published in W. C. Cheng et al. (1975) and, although ascribed to “K. M. Feng”, there is no suggestion that the descriptive material in the protologue was provided by K. M. Feng. Under Art. 46.5 of the ICN, this name should be attributed to K. M. Feng ex W. C. Cheng et al. but not to K. M. Feng alone. It has been claimed that the name is an illegitimate later homonym of one published by Beissner in 1901, but Beissner never accepted this name in any of his publications and so, under Art. 36.1, he did not validly publish an earlier homonym. *Cephalotaxuslanceolatus* was first validly published in W. C. Cheng et al. (1975).

The name *Cephalotaxuslanceolata* was described in Cheng et al. (1975: 86) and ascribed to K. M. Feng, but there was no indication in that publication that the diagnosis of the new species was provided by K. M. Feng. Likewise, in Cheng et al. (1978), “Flora Reipublicae Popularis Sinicae”, no author contribution by K. M. Feng was acknowledged. Consequently, under Art. 46.5 of the ICN (Turland et al. 2018), this name should be attributed to the authors of the publication and cited as “K. M. Feng ex W. C. Cheng et al.” or just “W. C. Cheng et al.”. As a result, the attribution of this name to K. M. Feng by Cheng (1983), Farjon (2010) and Lang et al. (2013a, 2013b) is incorrect.

Lang et al. (2013a, 2013b) considered *Cephalotaxuslanceolata* W. C. Cheng et al. to be an illegitimate later homonym because they thought that there was an earlier homonym, *C.lanceolata*Beissner (1901a). Therefore, they proposed the replacement name *C.talonensis* W. C. Cheng & L. K. Fu ex S. G. Lu & X. D. Lang taking up a manuscript name that W. C. Cheng & L. K. Fu and/or K. M. Feng had used on the original specimen. If Lang et al.’s claim was correct, then a replaced name would indeed be required.

I conducted a new investigation on the publications of Beissner (1901a, 1901b, 1909) and found that Beissner did not accept *C.lanceolata* as the name of a species in any of the three publications and so it is not validly published in any of them (Art. 36.1). Beissner (1909) treated *C.lanceolata* as a synonym of “*Cephalotaxusfortuneirobusta* hort.”, so clearly not accepting the name and the same is the case in the two 1901 publications: Beissner (1901b) wrote: “Nous devons dès lors regarder le *C.lanceolata* hort. comme une forme vigoureuse, multipliée par greffe, de *C.fortunei* Hook.” Translated to English as: I must therefore look at *C.lanceolata* hort. as a vigorous, graft-propagated form of *C.fortunei* Hook. Beissner (1901a) wrote: “Damit erledigt sich dann auch der zweite Punkt, dass *C.lanceolata* hört, nicht mit *C.griffithii* gleich ist, sondern als besonders langblättrige, üppige, durch Veredelung fixierte Form zu *C.fortunei* Hook, gehören dürfte.” This suggests that *C.lanceolata* hort. is not the same as *C.griffithii*, but should belong to *C.fortunei* Hook. as a particularly long-leaved, luxuriant form fixed by grafting.

As a conclusion, I consider that *C.lanceolata* should be ascribed to K. M. Feng ex W. C. Cheng et al. and that *C.lanceolata* K. M. Feng ex W. C. Cheng et al. is a legitimate name.

The taxonomic status of *C.lanceolata* has been in debate. The name was accepted in Cheng et al. (1978), Cheng (1983), Fu (1984), Huo (1986), Farjon (1998, 2010, 2017) and Fu et al. (1999) as that of a distinct species, but Silba (1990) treated it as a variety: C.fortuneivar.lanceolata (K. M. Feng ex W. C. Cheng et al.) Silba, which is also accepted by Eckenwalder (2009). Tripp (1995) considered *C.lanceolata* as a separate species from *C.griffithii* Hook. f. but Bisht et al. (2021), who thought the name illegitimate, treated it as a synonym of *C.griffithii*. Wang et al. (2022) conducted DNA barcoding research by sampling Chinese materials, and concluded that *C.lanceolata* represented a separate species lineage. Here I follow Tripp (1995), Farjon (1998, 2010, 2017), Fu et al. (1999) and Wang et al. (2022) and accept *C.lanceolata* as a separate species.

## ﻿Treatment

### ﻿ Cephalotaxus lanceolata

Taxon classificationPlantaePinalesCephalotaxaceae

K. M. Feng ex W. C. Cheng et al., Acta Phytotax. Sin. 13(4): 86 (1975)

C7DA175F-E96C-5BAD-8B66-E75D531DF41D

#### Homotypic synonym.

Cephalotaxusfortuneivar.lanceolata (W. C. Cheng et al.) Silba, Phytologia 68(1): 27 (1990); *Cephalotaxustalonensis* W. C. Cheng & K. M. Feng ex S. G. Lu & X. D. Lang, Bull. Bot. Res., Harbin 33(1): 5 (2013), nom. illeg.

#### Type.

**Figure 1.**
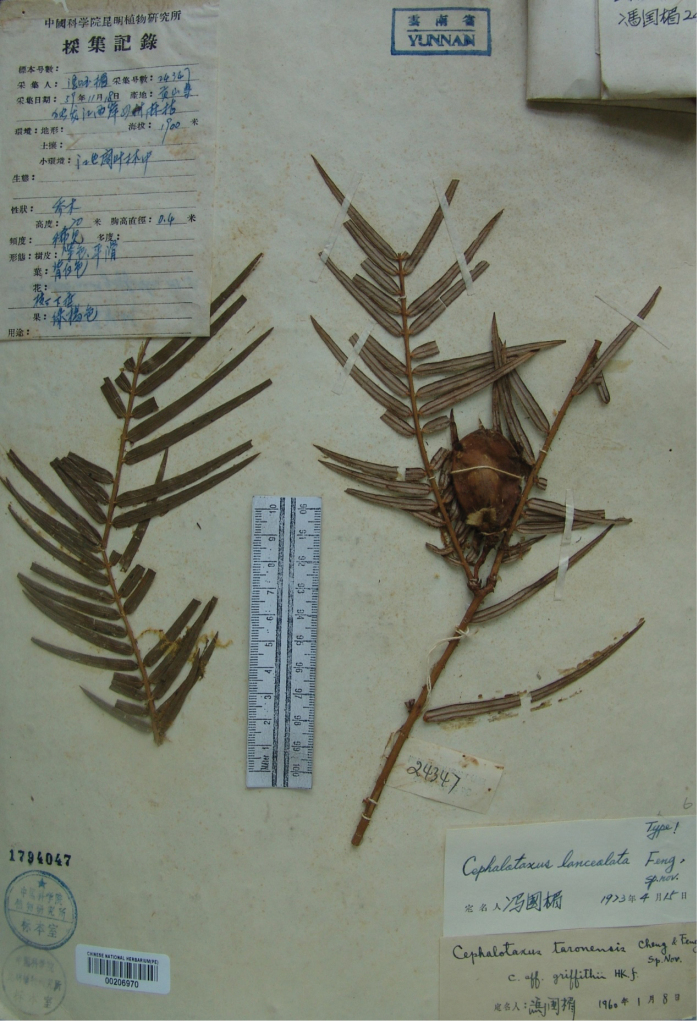
Holotype of *Cephalotaxuslanceolata* K. M. Feng ex W. C. Cheng et al. (PE00206970).

China (中国), Yunnan (云南), Gongshan Co. (贡山县), west of Dulong River, alt. 1900 m, in broad-leaved forests nearby the river, 18 Nov. 1959, *K. M. Feng* (冯国楣) *24347* (holotype: PE00206970, Fig. 1).

## Supplementary Material

XML Treatment for Cephalotaxus lanceolata

## References

[B1] Beissner L (1901a) Mitteilungen uber coniferen, die Taxales. Mitteilungen der Deutschen Dendrologischen Gesellschaft 10: 73–74.

[B2] Beissner L (1901b) Conifere de Chine, recoltes par le Rev, pere Joseph Giraldi dans le Shen-si septentrional et meridional. Bulletino della Societa botanica italiana 8: 357–359.

[B3] Beissner L (1909) Handbuch der Nadelholzkunde. Systematik, Beschreibung, Verwendung und Kultur der Freiland-Coniferen, ed. 2. Paul Parey, Berlin. 10.5962/bhl.title.21664

[B4] Bisht S Khuraijam JS Singh R (2021) Revisiting the taxonomy of the names *Cephalotaxusmannii* and *C.griffithii* (Taxaceae). Phytotaxa 501(1): 189–194. 10.11646/phytotaxa.501.1.10

[B5] Cheng WC (1983) Cephalotaxaceae. In: Cheng WC (Ed.) Silva Sinica (vol. 1). Beijing Forestry Publishing House, Beijing, 379.

[B6] Cheng WC Fu LK Cheng CY (1975) Gymnospermae Sinicae. Acta Phytotaxonomica Sinica 13: 56–89. 10.1016/0002-8703(75)90099-X

[B7] Cheng WC Fu LK Chao CS (1978) Cephalotaxales. In: Cheng WC Fu Lk (Eds) Flora Reipublicae Popularis Sinicae (vol.7). Science Press, Beijing, 424–426.

[B8] Eckenwalder JE (2009) Conifers of the World. The Complete Reference. Timber Press, Portland.

[B9] Farjon A (1998) World Checklist and Bibliography of Conifers. Royal Botanical Gardens at Kew, Richmond.

[B10] Farjon A (2010) A Handbook of the World’s Conifers (vol. 1). Brill, Leiden-Boston. 10.1163/9789047430629

[B11] Farjon A (2017) A Handbook of the World’s Conifers. 2^nd^ ed. Brill, Leiden-Boston. 10.1163/9789004324510

[B12] Fu LK (1984) A study on the genus *Cephalotaxus* Sieb. et Zucc. Acta Phytotaxonomica Sinica 22: 277–288.

[B13] Fu LK Li N Mill RR (1999) Cephalotaxaceae. In: Wu ZY Raven PH (Eds) Flora of China (vol.7). Science Press, Beijing & Missouri Botanical Garden, St. Louis, 85–88.

[B14] Huo SH (1986) Cephalotaxaceae. In: Wu CY (Ed.) Flora Yunnanica (vol.4). Science Press, Beijing, 109–111.

[B15] Lang XD Su JR Lu SG Zhang ZJ (2013a) A taxonomic revision of the genus *Cephalotaxus* (Taxaceae). Phytotaxa 84(1): 1–24. 10.11646/phytotaxa.84.1.1

[B16] Lang XD Su JR Lu SG (2013b) *Cephalotaxustalonensis* Cheng et Feng ex S. G. Lu et X. D. Lang, a new name of the family Cephalotaxaceae and its taxonomic status. Bulletin of Botanical Research 33: 4–6.

[B17] Silba J (1990) A supplement to the international census of the Coniferae. Phytologia 68: 27.

[B18] Tripp KE (1995) *Cephalotaxus*: The plum yews. Arnoldia 55: 25–39.

[B19] Turland NJ Wiersema JH Barrie FR Greuter W Hawksworth DL Herendeen PS Knapp S Kusber W-H Li D-Z Marhold K May TW McNeill J Monro AM Prado J Price MJ Smith GF (2018) International Code of Nomenclature for algae fungi and plants (Shenzhen Code) adopted by the Nineteenth International Botanical Congress, Shenzhen, China, July 2017. Regnum Vegetabile 159. Koeltz Botanical Books, Glashütten. 10.12705/Code.2018

[B20] Wang J Fu CN Mo ZQ Möller M Yang JB Zhang ZR Li DZ Gao LM (2022) Testing the complete plastome for species discrimination, cryptic species discovery and phylogenetic resolution in *Cephalotaxus* (Cephalotaxaceae). Frontiers in Plant Science 13: 768810. 10.3389/fpls.2022.768810 PMC9116380 35599857

